# Knee Lymphocutaneous Fistula Secondary to Knee Arthroplasty

**DOI:** 10.1155/2014/806164

**Published:** 2014-12-15

**Authors:** T. Pérez-de la Fuente, E. Sandoval, A. Alonso-Burgos, L. García-Pardo, C. Cárcamo, O. Caballero

**Affiliations:** ^1^Department of Plastic and Reconstructive Surgery, Fundación Jiménez Díaz, Avenida Reyes Católicos 2, 28040 Madrid, Spain; ^2^Department of Orthopedic Surgery, Fundación Jiménez Díaz, Avenida Reyes Católicos 2, 28040 Madrid, Spain; ^3^Department of Radiology, Fundación Jiménez Díaz, Avenida Reyes Católicos 2, 28040 Madrid, Spain

## Abstract

Lower limb lymphorrhea secondary to a surgical procedure is a rare but difficult-to-solve complication. In lower limb, this entity is frequently associated with vascular procedures around the inguinal area. We report on a case of a knee lymphocutaneous fistula secondary to a knee revision arthroplasty. To our knowledge, no previous reports regarding this complication have been published.

## 1. Introduction

The incidence of postoperative lymphocutaneous fistula ranges from 2% to 5% of all reconstructive vascular procedures of the lower limb. Uncontrolled lymphorrhea may lead to a lymph collection and breakdown of the surgical wound [1]. This condition increases morbidity, and secondary infection may be catastrophic in cases of associated prosthetic hardware. Lymphocutaneous fistulae after vascular procedures, especially around the inguinal region, have been widely reported, but, to the best of our knowledge, no reports on cases involving a knee arthroplasty have been previously published. The authors have obtained the patient's informed written comment for printing an electronic publication of the case report.

## 2. Case Report

**Figure 1:**
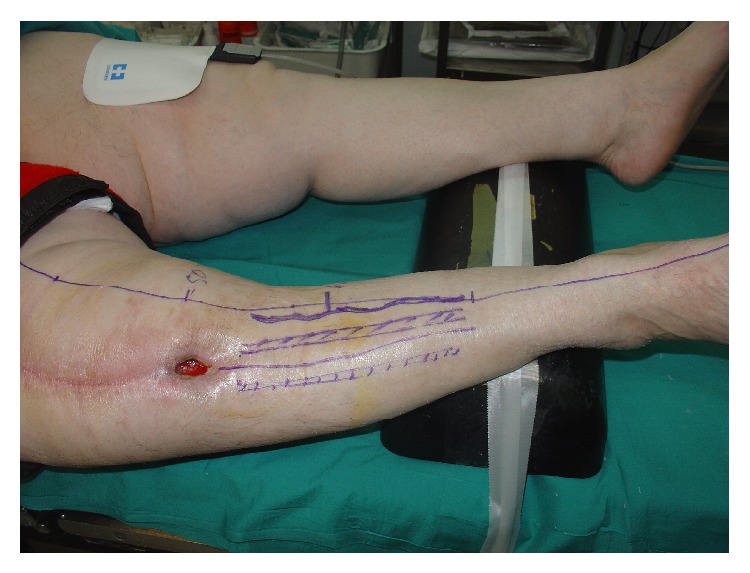
Knee wound secondary to a lymphocutaneous fistula.

A 72-year-old woman presented with a prosthetic joint infection after a revision knee arthroplasty, performed for a chronic primary arthroplasty infection through a standard anterior approach. A methicillin-resistant* Staphylococcus aureus* was isolated in conventional cultures from knee joint fluid. The surgical wound had a torpid evolution and needed in total three consecutive debridement processes and joint spacers (Figure 1).

After the third debridement, she developed a wound dehiscence, with an evident continuous transparent fluid drainage. Bacterial cultures of the fluid were negative and its analysis revealed the presence of glucose (86 gm/dL), proteins (2,8 gm/dL), LDH (5045,00 UI/dL), and white blood cells (1280/mm^3^) with 90% PMN, suggestive of lymph fluid.

**Figure 2:**
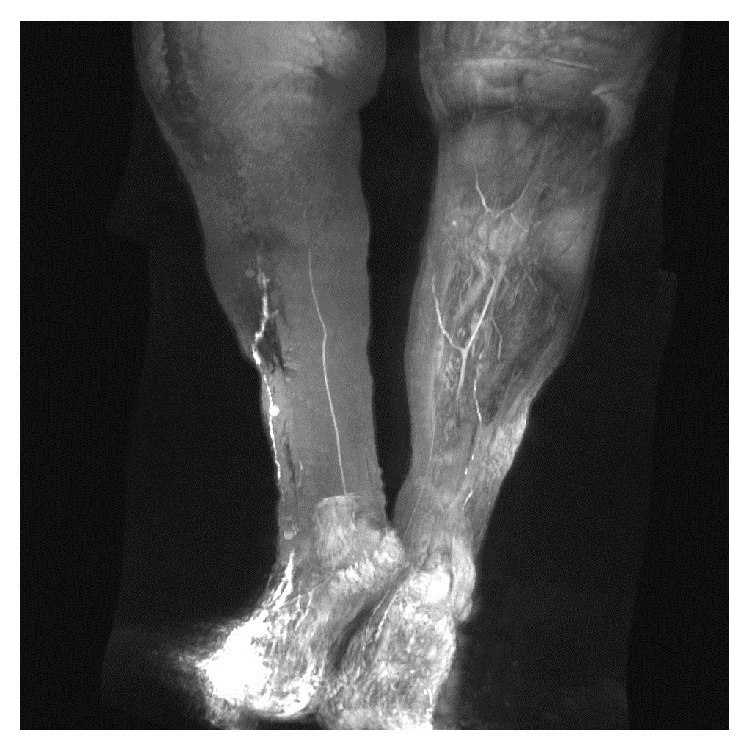
Two afferents superficial lymphatic vessels to the knee in MRL.

The first therapy was a compression bandage and leg rest, which resulted in no success. Therefore, a VAC therapy with continuous 75 mm Hg pressure was used for seven weeks. No wound closure was observed after that time and a persistent lymphocutaneous fistula was developed. Hence, a magnetic resonance lymphangiography (MRL) was performed. On the MRL images, the deep system remained unharmed, but two major lymphatic vessels of the superficial system were found to be directly afferent to the wound dehiscence (Figure 2).

**Figure 3:**
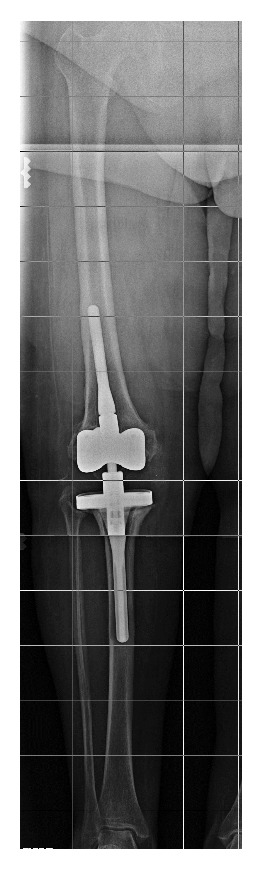
Hinged revision total knee arthroplasty after second stage replacement surgery.

Regarding the MRL results, a microsurgical lymphatic venous derivation was planned. Indocyanine green was used intraoperatively to identify lymphatic vessels, by performing an intradermal injection into the dorsal aspect of the four interdigital webs of the ipsilateral foot. During intraoperative exploration, no fluorescence was noted after the indocyanine green injection, probably due to the competence of the deep system. In contrast, two red lines corresponding clinically to a lymphangitis were observed on the skin along the anterior aspect of the leg, from the dorsum of the foot up to the knee wound. According to the MRL coordinates, these lines were the two major afferents lymphatic vessels. Secondarily, a new attempt with an injection of methylene blue in the dorsal aspect of the foot was carried out with no result and was also explained by the competence of the deep system. As a result, a dermic and subdermic lymphatic tissue from around the wound was ligated (in red on the skin) and a pedicle rotation flap was performed, to close the knee wound defect and to place the tension points out of the damaged knee scar line. During postoperation, no wound dehiscence or lymphorrhea occurred, and methylene blue clearance was seen through urine in a green color, a fact that suggested that the dye was really absorbed in a correct way. Ten months later, the patient underwent the second stage of total knee replacement with no skin incidence. During this time, she received antibiotics according to the sensitivity of the responsible bacteria (Figure 3).

## 3. Discussion

Treatment of lymphorrhea includes different types of management which range from nonsurgical to microsurgery procedures. To date, there are no international treatment guidelines or consensus on which is the most effective treatment [2]. Regarding nonsurgical treatment, nutritional approaches with low-fat diet with medium-chain triglycerides (MCT), enteral nutrition with a specialized formula, parenteral support, or some combination of them represent the first option. Long-chain triglycerides (LCT) are the main component of the fat obtained from diet. An average adult lymph flow contains 1500 mL of lymph with 70 grams of fat and 50 grams of proteins. Therefore, the consequences of lymph leakage are loss of fat and proteins. LCT absorption leads to a higher lymph flow and increases the fat and protein leakage. In opposition to LCT, MCT are water soluble, which makes them suitable for the treatment of this condition. In addition, MCT are directly absorbed by the portal venous system as fatty acids attached to the albumin, bypassing the enteric lymphatics. For these reasons, LCT restriction and MCT supplementation should help decreasing the lymph flow. Treatment modalities for secondary surgical lymphatic cutaneous fistulae are as follows. 

*Lymphocutaneous Fistula*

- Nonsurgical treatment includes
  - MCT diet, bed rest, and compressive dressings;
  - instillation sclerosing agents;
  - low-dose radiotherapy;
  - negative pressure wound therapy.
- Surgical treatment includes
  - lymphatic ligation dyed assisted;
  - lymphatic-venous anastomoses.

Somatostatin analogues (octreotide) have also been used to reduce volume and duration of lymphorrhea. The mechanism by which somatostatin analogues reduce lymphorrhea is still unknown. Somatostatin is a tetrapeptide hormone found among the nervous and the gastroenteropancreatic system. A direct effect of somatostatin on the lymph flow has only been observed in the gastrointestinal track; nevertheless, somatostatin receptors have been found in lymphatic tissues, within and outside of the gastrointestinal track [3]. Other treatments include instillation of doxycycline as a sclerosing agent into the lymphatic vessels [4]. After conservative treatment failure, external radiotherapy can be used. Doses less than 10 Gy are sufficient in more than 50% of cases to achieve clinical remissions in lymphocutaneous fistulas. Dietl et al. [5] observed quantitative reduction of inguinal lymphorrhea after radiotherapy (9 Gy total dose, 3 single doses) in 28 patients, allowing the drains to be removed within a median of 7 days after radiotherapy. Mayer et al. [6] treated 17 patients with lymphocutaneous fistula with total doses of 1–12 Gy, using single fractions 0,3–2 Gy. Thirteen patients showed a complete obliteration of the fistula, with total doses less than 3 Gy in nine cases.

Negative-pressure wound therapy has been also used, as described by Greer et al. [7]. This author demonstrated complete wound healing in lymphocutaneous fistula in two patients, after 16 days and 7 weeks, respectively. Posteriorly Abai et al. [8] reported on three cases with this therapy with a mean time of 14 days for cessation of lymphatic drainage.

Regarding surgical treatment, microsurgical procedures performing lymphatic-venous derivative drainage have recently become popular. Technically, it consists of several lymphatic-venous microanastomoses performed microsurgically by using a U-shaped stitch that connects the lymphatic vessel to the vein, reinforced with peripheral stitches. Dyes like methylene blue or indocyanine green are used to visualize the lymphatics and their drainage into the vein [9]. The use of indocyanine green as a contrast agent to visualize arteries, veins, and lymphatics vessels has increased in recent years. This technique has several advantages (absence of radiation, real-time monitoring) and also disadvantages (optimal ambient conditions, learning curve). When indocyanine green is injected in a human body, it rapidly bounds to plasma proteins, mainly high-density lipoproteins, and generates fluorescence in near infrared (845 nm center wavelength) which generates a light between 750 800 nm wavelength that can be captured by a camera [10, 11].

Knee swelling, wound dehiscence, and prosthetic joint infection are complications that usually occur following knee revision surgery; nevertheless, lymphocutaneous fistula is not seen so often. This complication might become really frustrating, as it delays the wound healing and prolongs the hospital stay.

In our case, many options of nonsurgical treatment were sequentially used. Firstly, MCT diet with restriction of LCT was followed by the patient with no result. Secondly, leg elevation and compression, followed by a negative pressure therapy, were used for 7 weeks showing no resolution of the symptoms. Therefore, surgery came up as the next treatment. We consider magnetic resonance lymphangiography (MRL) mandatory for the assessment of the lymphatic system and localization of the leak. The lymphatic system has two planes: a deep one anatomically located close to major vessels and a superficial one inside the dermis. The first one is dominant and is mainly responsible for the lymphatic drainage of the limb. Regarding our patient, the deep system remained normal, whether the superficial plane showed contrast media extravasation clearly visualized at the level of the knee, creating a lymphocutaneous fistula. We personally agree with Lohrmann's studies, in which MRL with intracutaneous injection of an extracellular paramagnetic contrast agent is considered to be a useful diagnostic imaging method for the evaluation of the lymphatic system. This imaging test is minimally invasive, lacks radiation, and bears no risks for the patient [12–14].

Up to this time, lymphoscintigraphy and intraoperative methylene blue staining have been used to describe the lymphatic anatomy in patients suffering from lymphocutaneous fistulae [15–17]. Lymphoscintigraphy has the disadvantage of using ionizing radiation and offering poor-resolution images comparing to MRL.

Regarding surgery, we initially planned a direct lymphatic-venous anastomose microsurgical reconstruction. However, after indocyanine green and methylene blue injection, lymphatic vessels were not identified, a fact that made direct reconstruction not possible. The competence of the deep lymphatic system in this case did not allow visualizing the superficial system. Tyndall et al. reported also that intraoperative methylene blue staining did not help to identify the leak sites in 10 patients [18].

A cutaneous reaction after indocyanine green injection was observed on skin, drawing two large red lines from dorsal aspect of the foot to wound dehiscence, which corresponded to the two superficial feeding lymphatic vessels on the MRL images. According to these findings, subcutaneous tissue was ligated including the superficial feeding lymphatics. A previous report on this technique was published by Schwartz et al. [19], who used a dye-system ligation which showed a clear and precise identification of the transected lymphatic vessels; the authors concluded that the use of isosulfan blue associated with the intraoperative examination allows for rapid and accurate information of the damage lymphatic vessels with no additional side effects.

## 4. Conclusions

Lymphatic damage should be in mind regarding failed knee reconstructive surgeries that are present with wound dehiscence or chronic swelling. Multiple underlying factors are involved, especially female gender, obesity, infection, and previous knee surgery. Special attention should be paid to specifically recognize this condition and solve it promptly, so that arthroplasty failure or further complications could be minimized.
